# Burnout and absenteeism: What impact on fitness for work?

**DOI:** 10.1192/j.eurpsy.2025.1566

**Published:** 2025-08-26

**Authors:** W. Ayed, M. Mersni, I. Youssef, N. Mechergui, H. Bensaid, G. Bahri, D. Brahim, M. Bani, N. Ladhari, A. Hakiri, I. Besbes, R. Ghachem

**Affiliations:** 1 Occupational medecine departement, Charles Nicolle Hospital; 2 Occupational medecine departement, Mongi Slim Hospital; 3 Occupational medecine departement, Habib Thameur Hospital; 4 Occupational medecine departement, Maternity and neonatal centre, Tunis; 5 Razi Hospital, Department B, Mannouba, Tunisia

## Abstract

**Introduction:**

Burnout is a prolonged response to chronic emotional and interpersonal stressors on the job and is defined by the three dimensions of exhaustion, cynicism, and inefficacy. Burnout has a major impact on employees’ health and the economy through absenteeism.

**Objectives:**

- To describe the socio-professional characteristics of patients suffering from burnout
- To Specify the impact of burnout on the medical fitness for work.

**Methods:**

Retrospective descriptive study of patients with burnout who consulted the occupational pathology and fitness for work department, during the period from 17 August 2022 to 14 September 2023, as part of a medical examination for long-term absence.

**Results:**

Of the 669 patients who presented for medical control, 222 (33.2%) suffered from burnout. The average age was 45.51±10.69 years. They were predominantly female, with a sex ratio (M/F) of 0.43. They belonged to the health (78.8%) and service (21.2%) sectors. The most common occupations were nurses (32.9%), manual workers (19.4%), and administrative staff (7.4%). The average length of time off work was 60.5±32.6 days. Medical decisions were to justify sick leave (73%) and return to work (24.8%).

**Conclusions:**

Burnout is responsible for absenteeism, which is a major source of expense. Prevention based on collaboration between the occupational physician and the psychologist is essential.

**Disclosure of Interest:**

None Declared

